# Clinical relevance of oncologic prognostic factors in the decision-making of pre-hepatectomy chemotherapy for colorectal cancer hepatic metastasis: the priority of hepatectomy

**DOI:** 10.1186/s12957-018-1322-9

**Published:** 2018-02-07

**Authors:** Kun-Ming Chan, Tsung-Han Wu, Yu-Chao Wang, Chen-Fang Lee, Ting-Jung Wu, Hong-Shiue Chou, Wei-Chen Lee, Jy-Ming Chiang, Jinn-Shiun Chen

**Affiliations:** 1 Division of Liver and Organ Transplantation Surgery, Department of General Surgery, Chang Gung Memorial Hospital at Linkou, Chang Gung University College of Medicine, 5 Fu-Hsing Street, Kwei-Shan District, Taoyuan City, 33305 Taiwan; 2 Department of General Surgery, Chang Gung Memorial Hospital at Linkou, Chang Gung University College of Medicine, 5 Fu-Hsing Street, Kwei-Shan District, Taoyuan City, 33305 Taiwan; 3 Department of Colorectal Surgery, Chang Gung Memorial Hospital at Linkou, Chang Gung University College of Medicine, 5 Fu-Hsing Street, Kwei-Shan District, Taoyuan City, 33305 Taiwan

**Keywords:** Colorectal cancer, Hepatic metastasis, Liver resection, Chemotherapy, Prognostic factors

## Abstract

**Background:**

Although liver resection (LR) provides the best chance of long-term survival for patients with colorectal cancer (CRC) hepatic metastasis, concerns regarding chemotherapy before liver resection remain unresolved.

**Methods:**

A retrospective review of patients who underwent curative LR for CRC hepatic metastasis between January 2008 and February 2016 was performed. Outcome relevance based on oncologic prognostic factors and chemotherapy prior to liver resection was assessed.

**Results:**

Patients who had received pre-hepatectomy chemotherapy for CRC hepatic metastasis and delayed liver resection had a worse outcome in terms of CRC recurrence following liver resection. The hazard ratio (HR) of pre-hepatectomy chemotherapy in patients with minor oncologic prognostic factors was 1.55 (confidence interval, CI = 1.07–2.26, *p* = 0.021) for CRC recurrence after liver resection for hepatic metastasis, whereas the HR of pre-hepatectomy chemotherapy was 1.34 (CI = 0.99–1.81, *p* = 0.062) for CRC recurrence in patients with multiple oncologic prognostic factors.

**Conclusion:**

The administration of pre-hepatectomy chemotherapy and delaying liver resection seems not to be an optimal strategy to provide a clinical benefit for patients with CRC hepatic metastasis. Hence, liver resection should be attempted without delay at the initial detection of CRC hepatic metastasis whenever possible.

## Background

Colorectal cancer (CRC) is a common malignancy, ranking in the top three most commonly diagnosed cancers worldwide [1]. It is estimated that up to 50% of CRC patients encounter hepatic metastasis during their disease course [2, 3]. Among those patients, 20–35% of patients with metastatic disease have the liver as their sole site of metastases, leading to liver resection as an optimal treatment for CRC with hepatic metastasis [4, 5]. Generally, liver resection with complete removal of the metastases has dramatically improved long-term survival, which ranges from 36 to 58% at 5 years and from 23 to 36% at 10 years [6–9]. Along with the advancement of anesthesia and progress of surgical approaches, the safety of liver surgery has improved remarkably [10–13]. As such, liver resection for hepatic metastasis from CRC has become a standard of clinical care for these patients in the current era.

However, CRC recurrence after liver resection for metastases remains a great concern, and the reported recurrent rates could be up to 60% [14, 15]. Despite advances in multimodal therapy and aggressive surgical approaches, recurrence is still encountered in certain populations of patients who have undergone liver resection for metastases. Specifically, patients with multiple oncological risk factors or invisible micrometastases on imaging are the group at high risk of developing CRC recurrence after liver resection. Currently, an effective treatment protocol that involves the multidisciplinary treatment in terms of the priority of therapeutic options, such as systemic chemotherapy and liver resection, remains unsettled. Particularly, the optimal time of liver resection for patients with multiple oncologic risk factors—the so-called borderline resectable metastases—is still uncertain. Thus, the purpose of this study was to review our experience with liver resection for patients with hepatic metastasis from CRC and to analyze patients’ outcomes based on the relevance of oncologic prognostic factors and neoadjuvant chemotherapy prior to liver resection.

## Methods

### Patients

A retrospective review of medical record from patients who had undergone liver resection (LR) for CRC hepatic metastasis between January 2008 and February 2016 at Chang Gung Memorial Hospital, Linkou Medical Centre, Taoyuan, Taiwan, was performed under approval of the institutional review board. This study was fully reviewed and endorsed by the internal review board of Chang Gung Memorial Hospital at Linkou (201700231B0), and informed consent from patient was waived due to the retrospective design of this study. A total of 476 patients including 324 men (68.1%) and 152 women (31.9%) were analyzed, and the median age of patients at the time of LR was 60.3 years (range, 28.8–88.0 years). Hepatic metastasis was scored by the defined oncologic risk factors including multiple metastases (≥ 5 nodules), size of largest metastasis > 5 cm in diameter, synchronous metastasis, lymph node-positive primary CRC, and high tumor marker levels with carcinoembryonic antigen (CEA) level > 60 ng/mL [4, 9, 16]. Accordingly, patients were categorized into two groups based on scores for oncologic prognostic factors [4]: group I, scores ≥ 2 indicating so-called borderline resectable or not optimally resectable metastases (*n* = 266); group II, scores < 2 (*n* = 210). The clinical characteristics in terms of prognostic factors and outcomes were analyzed for these two groups.

### Treatment of hepatic metastases

The treatment of CRC hepatic metastasis was determined by consensus obtained from the multidisciplinary committee of CRC, in which members comprise liver surgeons, proctologists, oncologists, diagnostic radiologists, and interventional radiologists. Treatment options were mainly determined by the balance among tumor characteristics and the patient’s physical condition and underlying liver status. The tumor status of CRC was thoroughly assessed using computed tomography (CT) scans from the neck to pelvic areas for all patients prior to surgery. Magnetic resonance imaging (MRI) and/or positron emission tomography (PET) or PET/CT were occasionally performed in selected patients if indicated to confirm occult metastasis. Generally, patients with concurrent unresectable extrahepatic metastases and anticipated inadequate remnant liver volume after LR were considered unsuitable for LR. Apart from that, the major concerns of LR were removal of all hepatic lesions with a curative intent and preservation of at least two contiguous hepatic segments with an adequate remnant liver volume and a sufficient vascular inflow and outflow. Based on intention of curative resection, the extent of LR was determined by pre-operative imaging study as well as confirmed by intra-operative ultrasonography to remove all hepatic metastasis. All metastatic nodules were completely removed by LR for all patients, and patients who had a combination of tumor ablation and LR during operation were not included in this study.

### Follow-up and chemotherapy

After liver resection, all patients were followed up at regular intervals at our clinic until death or the end of this study. During follow-up, clinical assessments including physical examination, serum laboratory tests, CEA measurement, and liver ultrasonography were performed 1 month after LR and every 3 months thereafter. Computed tomography (CT) and/or MRI were arranged on an annual basis or whenever CRC recurrence was suspected by aforementioned clinical assessments. Positron emission tomography (PET) or PET/CT was not routinely performed but was occasionally employed for patients who had equivocal conventional imaging studies to detect occult metastasis if indicated. Recurrence of CRC was defined by the histological proving of a lesion from either biopsy or surgical resection and/or the evidence of a newly developed lesion detected by cross-sectional imaging studies.

The administration of perioperative chemotherapy was mainly based on tumor characteristics indicating advance disease, genetic variant of primary CRC, patient’s physical condition, and availability of chemotherapeutic regimens. Briefly, neoadjuvant or preoperative chemotherapy was administered to patients who were considered initially not optimal for LR or unresectable by the treatment committee, or due to the patient’s preference for nonsurgical treatment. Postoperative adjuvant chemotherapy was usually recommended for all patients after LR unless a patient was unwilling to receive chemotherapy or their physical status was unsuitable for chemotherapy. The chemotherapeutic options were mostly fluorouracil, leucovorin, and a combination of irinotecan or oxaliplatin with/without bio-agents, such as bevacizumab and cetuximab, as appropriate. Capecitabine and pyrimidine analog capsules were prescribed for patients who had not received intravenous chemotherapeutic regimens when possible.

### Statistical analyses

Outcome measures included recurrence-free survival (RFS) and overall survival (OS). RFS was estimated from the date of LR to the date of detected CRC recurrence or the date of last follow-up if no CRC recurrence occurred, and OS was measured from the date of LR to the date of death or the date of last follow-up by the end of this study. Survival analysis was carried out according to the Kaplan-Meier method, and the log-rank test was used to compare values. Continuous variables were compared by using Student’s *t* test, and categorical variables were compared by *χ*^2^ test as appropriate. Cox proportional hazards models were used to calculate the crude hazard ratios (HRs) and 95% confidence intervals (95% CIs). Subsequently, the adjusted HRs were obtained after adjustment of significant factors acquired from subgroup analysis. Statistical analyses were performed by using SPSS 20.0 statistical software (SPSS, Inc., Chicago, IL, USA) for Windows. A *p* value of < 0.05 was considered statistically significant.

## Results

### Clinical features of patients

Generally, the majority of patients (*n* = 433) had less than 5 metastatic nodules, which accounted for 90.9% of patients in the current study. The median metastatic nodules was 2 (range, 1–17 nodules), and the median size of maximum metastatic nodule was 3.0 cm (range, 0.3–14.5 cm) for all patients. After LR, the median follow-up time for all patients was 26.1 months, ranging from 0.03 to 95.7 months. Overall, 299 patients (62.8%) encountered CRC recurrence after LR, and 177 (37.2%) patients had no CRC recurrence by the date of last follow-up or the end of this study. There were four deaths during hospitalization for surgical treatment, which turned into 0.8% surgical mortality for all patients. Meanwhile, 184 (38.7%) patients died during the follow-up period, in which 175 (36.8%) patients died of CRC and 9 (1.9%) died of diseases other than CRC. Two hundred eighty-eight (60.5%) patients were still alive by the end of the study, including 164 (34.5%) patients who were CRC-free and 124 (26.1%) patients with recurrent CRC. Table 1 summarizes the clinical features of the patients who underwent LR for CRC hepatic metastasis and the comparison of two groups categorized on the basis of oncologic risk scores. There were significant differences in terms of the five oncologic risk factors between the two groups. Additionally, patients with multiple oncologic risk factors (scores ≥ 2) had a higher ratio of CRC recurrence (*p* = 0.004) and a lower ratio of CRC-free status (*p* = 0.026) after LR as compared with the other group.

**Table 1:** Clinicopathologic characteristics of patients undergoing liver resection for colorectal cancer hepatic metastasis

Characteristics	Oncologic scores ≥ 2 *n* = 266 (%)	Oncologic scores < 2 *n* = 210 (%)	*p* value
Age (years), median (range)	60 (29–88)	61 (29–88)	0.503
Gender			0.317
Male	176 (66.2)	148 (70.5)	
Female	90 (33.8)	62 (29.5)	
Primary tumor location			0.776
Right colon	43 (16.2)	36 (17.1)	
Left colon	223 (83.8)	174 (82.9)	
Metastatic types			< 0.0001
Synchronous	225 (84.6)	46 (21.9)	
Metachronous	41 (15.4)	164 (78.1)	
Serum CEA			< 0.0001
< 60 ng/mL	201 (75.6)	202 (96.2)	
≥ 60 ng/mL	65 (24.4)	8 (3.8)	
Maximum tumor size			< 0.0001
< 5 cm	178 (66.9)	200 (95.2)	
≥ 5 cm	88 (33.1)	10 (4.8)	
Tumor number			< 0.0001
< 5 nodules	226 (85.0)	207 (98.6)	
≥ 5 nodules	40 (15.0)	3 (1.4)	
Primary lymph node			< 0.0001
Positive	228 (85.7)	93 (44.3)	
Negative	38 (14.3)	117 (55.7)	
CRC recurrence after liver resection.			0.004
Yes	182 (68.4)	117 (55.7)	
No	84 (31.6)	93 (44.3)	
Patient’s final status			0.026
Alive and CRC-free	75 (28.2)	89 (42.4)	
Alive with recurrent CRC	77 (28.9)	47 (22.4)	
Dead of CRC	105 (39.5)	70 (33.3)	
Dead of other causes	6 (2.3)	3 (1.4)	
Surgical mortality	3 (1.1)	1 (0.5)	

*CRC* colorectal cancer

### Patient outcome

**Fig. 1:**
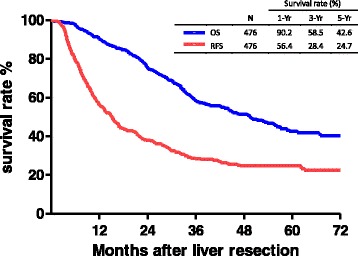
Kaplan-Meier cumulative recurrence-free and overall survival curves of patients with colorectal cancer (CRC) hepatic metastasis after liver resection

**Fig. 2:**
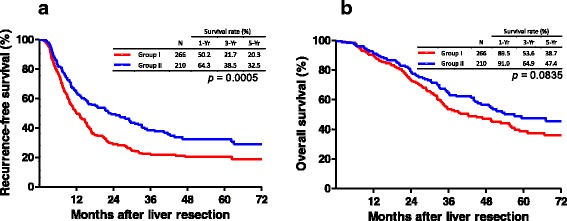
Comparison of Kaplan-Meier cumulative survival curves based on oncologic prognostic factors. **a** Recurrence-free survival. **b** Overall survival

During the follow-up period, the median time of CRC recurrence after LR was 15.1 months, and the 1-, 3-, and 5-year RFS rates were 56.4, 28.4, and 24.7%, respectively (Fig. 1). The median time of survival for all patients after LR was 49.4 months, and the 1-, 3-, and 5-year OS rates were 90.2, 58.5, and 42.6%, respectively (Fig. 1). A comparison of RFS and OS curves according to the oncologic scores is illustrated in Fig. 2. The RFS curve of patients with oncologic scores < 2 (1, 3, and 5 years; 64.3, 38.5, and 32.5%, respectively) was considerably better than that of patients with oncologic scores ≥2 (1, 3, and 5 years; 50.2, 21.7, and 20.3%, respectively) (Fig. 2a, *p* < 0.0005). However, there was no significant difference in the cumulative OS curves between the two groups, in which the 1-, 3-, and 5-year survival rates were 89.5, 53.6, and 38.7% (oncologic scores ≥ 2) versus 91.0, 64.9, and 47.4% (oncologic scores < 2), respectively (Fig. 2b, *p* = 0.0835).

### Chemotherapy before liver resection

Overall, 183 patients (38.4%) had received chemotherapy prior to liver resection. The clinical characteristics of patients based on whether pre-hepatectomy chemotherapy was performed or not is summarized in Table 2. The majority of clinical features were not significantly different in subgroup analysis, but patients who had received pre-hepatectomy chemotherapy had a younger age and more tumor nodules as compared with patients who had no chemotherapy before liver resection in both groups.

**Table 2:** The clinical characteristics of patients based on whether pre-hepatectomy chemotherapy was performed or not

	Oncologic scores ≥ 2			Oncologic scores < 2		
Characteristics	Pre-hepatectomy chemotherapy		*p* value	Pre-hepatectomy chemotherapy		*p* value
	Yes *N* = 104 (%)	No *N* = 162 (%)		Yes *N* = 79 (%)	No *N* = 131 (%)	
Age (years), median (range)	58 (29–86)	61 (29–88)	0.034	59 (29–84)	62 (34–88)	0.017
Gender			0.201			0.601
Male	64 (61.5)	112 (69.1)		54 (68.4)	94 (71.8)	
Female	40 (38.5)	50 (30.9)		25 (31.6)	37 (28.2)	
Primary tumor location			0.337			0.336
Right colon	14 (13.5)	29 (17.9)		11 (13.9)	25 (19.1)	
Left colon	90 (86.5)	133 (82.1)		68 (86.1)	106 (80.9)	
Type of initial hepatic metastases			0.167			0.353
Synchronous	84 (80.8)	141 (87.0)		20 (25.3)	26 (19.8)	
Metachronous	20 (19.2)	21 (13.0)		59 (74.7)	105 (80.2)	
Serum CEA (ng/mL), median (range)	11.1 (0.5–16,259)	13.4 (0.5–2954.0)	0.190	7.0 (0.6–589.4)	9.4 (0.9–170.5)	0.849
Maximum tumor (cm), median (range)	3.5 (0.6–14.5)	3.3 (0.4–12.9)	0.316	2.5 (0.8–10.0)	2.5 (0.3–9.4)	0.813
Tumor number, median (range)	3.0 (1.0–17.0)	1.5 (1.0–11.0)	0.002	2.0 (1.0–7.0)	1.0 (1.0–4.0)	0.024
Primary lymph node			0.959			0.387
Positive	89 (85.6)	139 (85.8)		38 (48.1)	55 (42.0)	
Negative	15 (14.4)	23 (14.2)		41 (51.9)	76 (58.0)	

**Fig. 3:**
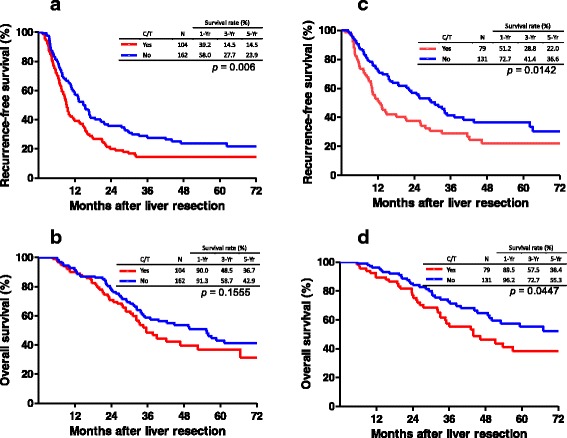
Cumulative survival curves of patients who underwent liver resection for hepatic metastasis according to oncologic prognostic factors and pre-hepatectomy chemotherapy. **a**, **b** Comparison of outcomes based on pre-hepatectomy chemotherapy for group I patients (oncologic prognostic factors ≥ 2). **c**, **d** Comparison of outcomes based on pre-hepatectomy chemotherapy for group II patients (oncologic prognostic factors < 2). RFS recurrence-free survival, OS overall survival

The outcomes in terms of RFS and OS according to whether chemotherapy had been administered before liver resection were further analyzed. Among patients with chemotherapy before liver resection, a worse RFS curve was observed as compared with that of patients without chemotherapy before liver resection in both groups. The 1-, 3-, and 5-year RFS rates were 39.2, 14.5, and 14.5%, respectively, for patients with chemotherapy before liver resection, and 58.0, 27.7, and 23.9%, respectively, for patients without chemotherapy before liver resection in group I (Fig. 3a, *p* = 0.006). However, the cumulative OS was not significantly different between patients with and without chemotherapy before liver resection in group I, in which the cumulative 1-, 3-, and 5-year OS rates were 90.0, 48.5, and 36.7%, versus 91.3, 58.7, and 42.9%, respectively (Fig. 3b, *p* = 0.1555). With regard to group II, the RFS curve of patients without chemotherapy before liver resection was significantly better than that of patients with chemotherapy before liver resection, in which the 1-, 3-, and 5-year RFS rates were 72.7, 41.4, and 36.6% versus 51.2, 28.8, and 22.0%, respectively (Fig. 3c, *p* = 0.0142). Similarly, the cumulative OS of patients without chemotherapy before liver resection was significantly better than that of patients with chemotherapy before liver resection, in which the 1-, 3-, and 5-year OS rates were 96.2, 72.7, and 55.3% versus 89.5, 57.5, and 38.4%, respectively (Fig. 3d, *p* = 0.0447).

**Fig. 4:**
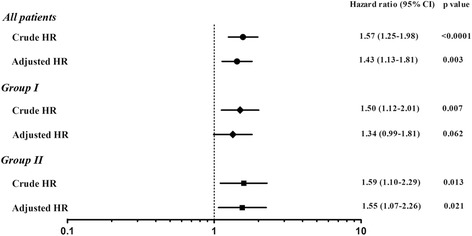
Risk analysis of hazard ratios for colorectal cancer recurrence after liver resection of hepatic metastasis associated with the use of pre-hepatectomy chemotherapy

The risk of post-hepatectomy CRC recurrence related to pre-hepatectomy chemotherapy is illustrated in Fig. 4, and the result shows that the risk of CRC recurrence was significantly associated with the administration of pre-hepatectomy chemotherapy in both groups. The crude HRs of CRC recurrence in terms of pre-hepatectomy chemotherapy for groups I and II were 1.50 (CI = 1.12–2.01, *p* = 0.007) and 1.59 (CI = 1.10–2.29, *p* = 0.013), respectively. After adjustment for age and number of metastatic nodules, which had shown statistical difference in subgroup analysis as shown in Table 2, the adjusted HRs for CRC recurrence were 1.34 (CI = 0.99–1.81, *p* = 0.062) for group I and 1.55 (CI = 1.07–2.26, *p* = 0.021) for group II.

## Discussion

Liver resection has been the standard practice for patients with hepatic metastases from colorectal cancer, and it currently offers the best chance of long-term survival and potential cure for patients with CRC. With the implementation of multidisciplinary treatment, the long-term outcome of patients with CRC hepatic metastasis has shown remarkable improvements over recent decades [9, 17, 18]. As it is one of the major lethal malignancies, extensive research on the optimal strategy for treatment of hepatic metastasis from CRC is always in demand. In this study, we found that waiting to administer chemotherapy before liver resection and delaying liver resection for CRC hepatic metastasis is not an optimal strategy, nor does it offer a clinical benefit to patients. However, the optimal timing of liver resection for patients with multiple oncologic risk factors that presumably would lead to potential CRC recurrence after liver resection remains uncertain, and it could rely on the surgeon’s decision based on experience.

Numerous reports have previously shown that several key prognostic factors would affect the outcomes of patients who undergo LR for hepatic metastasis from CRC [4, 9, 16, 19]. Of those factors, liver metastases characterized by metastases ≥ 5 nodules, size of largest metastasis > 5 cm in diameter, synchronous metastasis, lymph node-positive primary CRC, and high tumor marker levels are most often involved. Generally, patients who have more than one of the poor prognostic factors listed above at the time of liver metastasis detection are likely to develop early CRC recurrence, and they are usually considered not optimally resectable—also termed “borderline resectable metastases” [4]. Indeed, the cumulative CRC recurrence of patients with multiple oncologic risk factors was significantly higher than that of other patients in this study.

With regard to pre-operative chemotherapy, administration of chemotherapy before liver resection is usually considered in two settings: downsizing chemotherapy with the aim of making patients who have unresectable liver metastasis eligible for liver resection and neoadjuvant chemotherapy with the aim of improving long-term outcome by reducing post-hepatectomy cancer relapse for patients who have resectable liver metastasis. As such, for patients with unresectable liver metastasis, there is no doubt that chemotherapy is the only option, and liver resection could be considered if appropriate afterward. However, in cases of resectable liver metastasis, the administration of chemotherapy before liver resection remains arguable. By the time period of chemotherapy, tumor progression could be encountered and possibly turned into unresectable status if metastatic lesion is not susceptible to chemotherapy. Apart from that, there is no definitive protocol regarding regimens, and courses should be given as a neoadjuvant chemotherapy so far. Therefore, recent studies mostly did not recommend neoadjuvant chemotherapy for patients with upfront resectable liver metastasis due to a lack of benefit [20–22].

As shown in the present study, patients who received chemotherapy before liver resection had a shorter disease-free interval than those without pre-hepatectomy chemotherapy. However, patients who had received chemotherapy before liver resection were mostly characterized by a younger age and a higher number of hepatic metastases. Hence, it is possible to argue that patients who were subjected to pre-hepatectomy chemotherapy had naturally more severe hepatic metastasis than other patients in the study. As a result, patients who had received chemotherapy before liver resection had a poor outcome in terms of RFS. Nevertheless, further detailed analysis after adjustment of parameters showed a remarkable result. In line with previous reports, pre-hepatectomy chemotherapy for patients in group II (< 2 oncologic factors) would increase the risk of CRC recurrence after liver resection of metastasis, indicating that liver resection should not be delayed for patients with resectable metastasis and minor oncologic risk factors.

However, the risk of CRC recurrence was not affected by whether or not pre-hepatectomy chemotherapy was administered in group I patients (≥ 2 oncologic factors) according to the result of adjusted HR analysis. The data indicate that the timing of liver resection remains uncertain for high-risk patients, suggesting the crucial role of the liver surgeon’s experience in dealing with CRC hepatic metastasis for such patients. As long as the metastatic lesion is technically resectable, liver resection with a curative intent should be attempted under the prerequisite of preserving adequate future remnant liver volume. Nonetheless, certain populations of patients, such as those with multiple metastasis with a tumor number > 5, would ultimately recur even after curative resection of liver metastasis [14, 23]. Although neoadjuvant chemotherapy had been proposed as a selection tool for high-risk patients [24, 25], the presence of multiple metastasis should not be a contraindication of liver resection because of the absence of other alternative treatments associated with long-term survival as well as potential cures for the disease.

Additionally, potential concerns regarding the prolonged administration of chemotherapy before liver resection should also be mentioned. Although chemotherapy could downsize the metastatic tumor burden in a good site, it may induce many histologic changes of the liver parenchyma, including steatohepatitis, sinusoidal obstruction, and vascular injury, which would lead to an increased postoperative complication on the other hand [26–28]. Moreover, a study on major liver resection for CRC hepatic metastasis showed that irinotecan-induced steatohepatitis was associated with the risk of postoperative mortality, including death from liver failure [28]. The precise mechanisms of chemotherapy-induced liver injury remain unclear, and future development in terms of a better understanding of these mechanisms may possibly help counter the effects of chemotherapy on the liver.

Interestingly, the long-term overall survival was not significantly affected by the utilization of pre-hepatectomy chemotherapy in group I of this study. A theoretical explanation of this phenomenon could be that the adjuvant chemotherapy after liver resection may not only prevent CRC recurrence but also potentially extend patient’s survival. Apart from that, the current aggressive approach of multidisciplinary treatments including repeat liver resection, modern chemotherapy, and so on could also prolong patients’ survival even after CRC recurrence following liver resection [14, 29–31]. Therefore, a predicted high risk of cancer recurrence following hepatectomy for CRC hepatic metastasis should not be used as an exclusion criterion for liver resection.

## Conclusions

In summary, patients with multiple oncologic risk factors are certainly embracing a higher risk of CRC recurrence after liver resection as compared with other patients. Generally, the choice of therapeutic strategy should be individualized on the basis of balance against resectability of tumor status, extent of liver resection, and host condition for patients with CRC hepatic metastasis. As shown in this study, the administration of pre-hepatectomy chemotherapy for CRC hepatic metastasis seems to confer no clinical benefit in terms of preventing cancer recurrence after liver resection. Chemotherapy before liver resection is definitively not necessary for patients with low-risk hepatic metastasis. Although pre-hepatectomy chemotherapy remains controversial for high-risk hepatic metastasis, the result could also be interpreted as liver resection without chemotherapy is a logical strategy for these patients whenever possible. However, the optimal integration of chemotherapy with liver resection, including the use of pre-operative neoadjuvant chemotherapy, remains to be determined.

## Abbreviations

CEA: Carcinoembryonic antigen
CI: Confidence interval
CRC: Colorectal cancer
CT: Computed tomography
HR: Hazard ratio
LR: Liver resection
OS: Overall survival
PET: Positron emission tomography
RFS: Recurrence-free survival
